# Estimating Gestational Age in Late Presenters to Antenatal Care in a Resource-Limited Setting on the Thai-Myanmar Border

**DOI:** 10.1371/journal.pone.0131025

**Published:** 2015-06-26

**Authors:** Kerryn A. Moore, Julie A. Simpson, Kyla H. Thomas, Marcus J. Rijken, Lisa J. White, Saw Lu Moo Dwell, Moo Kho Paw, Jacher Wiladphaingern, Sasithon Pukrittayakamee, François Nosten, Freya J. I. Fowkes, Rose McGready

**Affiliations:** 1 Centre for Epidemiology and Biostatistics, Melbourne School of Population and Global Health, The University of Melbourne, Melbourne, Victoria, Australia; 2 Macfarlane Burnet Institute of Medical Research, Melbourne, Victoria, Australia; 3 Shoklo Malaria Research Unit, Mahidol-Oxford Tropical Medicine Research Unit, Faculty of Tropical Medicine, Mahidol University, Mae Sot, Thailand; 4 University Medical Centre Utrecht, Utrecht, The Netherlands; 5 Mahidol-Oxford Tropical Medicine Research Unit, Faculty of Tropical Medicine, Mahidol University, Bangkok, Thailand; 6 Centre for Tropical Medicine and Global Health, Nuffield Department of Medicine, University of Oxford, Oxford, United Kingdom; 7 Department of Epidemiology and Preventive Medicine and Department of Infectious Diseases, Monash University, Melbourne, Victoria, Australia; University Hospital Basel, SWITZERLAND

## Abstract

Estimating gestational age in resource-limited settings is prone to considerable inaccuracy because crown-rump length measured by ultrasound before 14 weeks gestation, the recommended method for estimating gestational age, is often unavailable. Judgements regarding provision of appropriate obstetric and neonatal care are dependent on accurate estimation of gestational age. We determined the accuracy of the Dubowitz Gestational Age Assessment, a population-specific symphysis-fundal height formula, and ultrasound biometry performed between 16 and 40 weeks gestation in estimating gestational age using pre-existing data from antenatal clinics of the Shoklo Malaria Research Unit on the Thai-Myanmar border, where malaria is endemic. Two cohorts of women who gave birth to live singletons were analysed: 1) 250 women who attended antenatal care between July 2001 and May 2006 and had both ultrasound crown-rump length (reference) and a Dubowitz Gestational Age Assessment; 2) 975 women attending antenatal care between April 2007 and October 2010 who had ultrasound crown-rump length, symphysis-fundal measurements, and an additional study ultrasound (biparietal diameter and head circumference) randomly scheduled between 16 and 40 weeks gestation. Mean difference in estimated newborn gestational age between methods and 95% limits of agreement (LOA) were determined from linear mixed-effects models. The Dubowitz method and the symphysis-fundal height formula performed well in term newborns, but overestimated gestational age of preterms by 2.57 weeks (95% LOA: 0.49, 4.65) and 3.94 weeks (95% LOA: 2.50, 5.38), respectively. Biparietal diameter overestimated gestational age by 0.83 weeks (95% LOA: -0.93, 2.58). Head circumference underestimated gestational age by 0.39 weeks (95% LOA: -2.60, 1.82), especially if measured after 24 weeks gestation. The results of this study can be used to quantify biases associated with alternative methods for estimating gestational age in the absence of ultrasound crown-rump length to inform critical clinical judgements in this population, and as a point of reference elsewhere.

## Introduction

Accurate determination of gestational age (GA) is essential for the provision of appropriate obstetric and neonatal care, including treatment of infections during pregnancy with drugs that may be contraindicated in the first trimester, detection of growth restriction and post-term pregnancies (≥42 weeks gestation), provision of antenatal corticosteroids during preterm labour, and decisions regarding whether to administer or withhold intensive care to extremely premature infants [1–4]. Fetal crown-rump length (CRL) measured by ultrasound between 7^+0^ and 13^+6^ weeks gestation is the recommended method for precise dating of spontaneously conceived pregnancies [5]. Beyond 14 weeks, ultrasound up to 24 weeks is the upper recommended limited for accurate dating using other fetal biometry measurements including head circumference (HC) and biparietal diameter (BPD) [5]. However, in resource-limited settings GA assessment is prone to inaccuracy. While several publications have demonstrated successful sonography in resource-limited settings, quality routine ultrasound is rarely available [6–8]. Where ultrasound is available, late attenders to antenatal care or birth centres present dating issues in all settings because ultrasound biometry is less accurate and less precise when measured later during pregnancy [9–11]. Therefore, estimating gestational age in the absence of CRL biometry is a problem of global significance.

Prior to ultrasound, various alternative methods were used to estimate GA. These methods are still widely practiced in resource-limited settings where ultrasound is unavailable, and in late presenters. Symphysis-pubis fundal height (SFH) measurements are commonly taken during antenatal care, and are used as a simple and inexpensive method of estimating GA from SFH growth charts [12]; a formula for estimating GA from at least three SFH measurements specific to this study population has been developed and is accurate to ±2 weeks [13]. Additionally, several clinical methods (requiring some technical expertise but little equipment or expenditure), such as the Ballard or the Dubowitz methods of GA assessment utilize external and neurological criteria of the newborn to determine GA at birth [14,15]. GA is also commonly calculated from the first day of the last menstrual period (LMP), but LMP is less well recalled in late attenders [16–19], and determination of LMP can be impeded by low literacy rates and cultural factors [7,8].

Accurate GA assessment is of particular significance in malaria endemic areas as the adverse maternal and fetal effects of exposure to malaria or antimalarial drugs used for treatment may be modified by gestation [3,20,21]. Additionally, although all methods of estimating GA will have a margin of error, large and systematic measurement error will lead to misclassification of adverse birth outcomes such as preterm birth, small for gestational age, intrauterine growth restriction, spontaneous abortion and stillbirth; misclassification will bias associations between exposure to malaria and antimalarial drugs during pregnancy and adverse birth outcomes. Hundreds of millions of pregnancies occur in resource-limited settings every year, including 125 million pregnancies at risk of malaria, where reliance on less accurate dating methods is common [22,23]. Therefore, determining the relative accuracy of alternative methods for estimating GA is vitally important to inform clinical judgements in obstetric and neonatal care and in epidemiological research of malaria in pregnancy.

We sought to determine the accuracy of the Dubowitz method, the SFH formula, and HC and BPD biometry measured between 16 and 40 weeks gestation in estimating newborn GA in a population of migrants and refugees on the Thai-Myanmar border attending antenatal clinics of the Shoklo Malaria Research Unit (SMRU), with reference to CRL biometry. Additionally, we sought to compare the accuracy of the Dubowitz method, the SFH formula, and HC biometry measured after 24 weeks, which is of particular clinical interest at SMRU because over one-third of women present late for antenatal care. To date, the accuracy of HC and BPD biometry has not been determined over birthweight-for-GA Z-score, newborn GA, and gestation time of biometry measurement. Similarly, the accuracy of the Dubowitz method and the SFH formula has not been compared to HC biometry measured after 24 weeks to determine which method is most accurate in late presenters. Furthermore, the accuracy of these methods has not been determined across newborn parameters that are known in the absence of CRL biometry, such as newborn GA estimated using alternative methods and birthweight-for-GA Z-score calculated from GA estimated using alternative methods. We have provided simple regression equations that will help clinicians assess gestational age in practice.

## Methods

### Study site and population

SMRU provides healthcare to refugees and migrants on the Thai-Myanmar border, including weekly screening for malaria in pregnant women due to a lack of other effective preventive measures in this area [24]. SMRU has been collecting longitudinal data of pregnant women presenting to antenatal care since 1986 representing, to the best of our knowledge, the largest longitudinal dataset of malaria in pregnancy to date. Methods for estimating GA at SMRU clinics have evolved over time, and these changes need to be considered when analysing maternal and newborn data from this 28-year period. Monthly SFH measurement was the predominant method for determining GA until 1992. Between 1992 and 1994 there was a gradual transition from SFH to the Dubowitz Gestational Age Assessment, though SFH continued to be routinely collected. Ultrasound was introduced in 2001 and became routine in 2002, after which Dubowitz exams were only performed on newborns whose mother hadn’t received timely ultrasound assessments (i.e. before 24 weeks gestation). Although LMP has been routinely collected in this population, many women (more than two-thirds) are unable to recall the date due to low literacy rates and unfamiliarity with Gregorian calendars [7].

SMRU ultrasound practice has also evolved over time, and is informed by the British Medical Ultrasound Society (BMUS) guidelines and local conditions. All women are encouraged to attend the antenatal clinic as early as possible. At the first visit, ultrasound is used to date pregnancies using CRL biometry between 7^+0^ and 13^+6^ weeks gestation (or between 7^+0^ to 10^+6^ weeks in the early years of ultrasound practice at SMRU, as CRL estimates between 11^+0^ and 13^+6^ weeks gestation were avoided to reduce error associated with a flexed fetus, which requires ultrasonographers to overcome a learning curve). For women presenting between 14^+0^ and 23^+6^ weeks gestation, BPD was used until 2007, after which HC became the preferred biometric for dating after 14 weeks [25]. The Robinson and Fleming formula is used for estimating GA from CRL biometry [26], the Altman and Chitty formula for estimating GA from HC biometry [25,27], and the formula of Hadlock *et al* is used for estimating GA from BPD biometry [16].

The equipment and quality control of the sonographers at SMRU have been detailed previously [1,7]. Associate Professor Lily Dubowitz introduced the Dubowitz gestational age assessment in 1994 and a quality control program was established in 1995 [28]. The staff involved in the Dubowitz assessment of gestational age were initially quality controlled against Associate Professor Dubowitz personally, and later against a series of test cards at six-monthly intervals. Details of SFH measurement at SMRU have also been detailed previously [13].

### Study design

Data from two cohorts were analysed for this study. First, the Dubowitz Gestational Age Assessment was compared to ultrasound CRL using routinely collected data on women who attended SMRU clinics between July 2001 and May 2006. Data were obtained from a de-identified SMRU database of Dubowitz scores. Inclusion criteria were: normal (as determined from a newborn exam for congenital abnormalities), live born, singletons; a complete Dubowitz score sheet filled out within 72 hours of a cephalic vaginal or vacuum delivery (women requiring caesarean are referred to hospital); and a CRL measurement of 10-41mm (corresponding to 7^+0^ to 10^+6^ weeks gestation). Pre- and post-term newborns were disproportionately selected to comprise 30% of the total sample in order to look at the extremes of gestation, where the Dubowitz Gestational Age Assessment was clinically suspected to be most inaccurate. Therefore, preterm (<37 weeks) and post-term (≥42 weeks) newborns (based on CRL estimates) were manually selected until records meeting the selection criteria were exhausted (n = 75). Then, records of term newborns were randomly selected until the total sample size reached 250 (n = 175).

Second, ultrasound HC and BPD measured after 14 weeks were compared to ultrasound CRL. Previously published data from 975 women attending the SMRU antenatal clinic at Maela refugee camp who participated in a study on the quality of ultrasound biometry between April 2007 and October 2010 was used [1]. Briefly, women who had an early CRL measurement of 10–80 mm (corresponding to 7^+0^ and 13^+6^ weeks gestation) were randomly assigned to receive one additional study scan between 16 and 40 weeks gestation, at which HC and BPD were measured twice by trained ultrasonographers blinded to the expected GA determined from CRL biometry [1]. Mother-newborn pairs that had an unknown outcome, GA below the viability cut-off of 28 weeks, resulted in stillbirth, or were complicated by serious infectious diseases (e.g. malaria) before the second ultrasound scan were excluded. Unlike the Dubowitz method cohort, women were recruited prospectively at antenatal care, so pre- and post-term newborns were not disproportionately selected. At least three symphysis-fundal height measurements were also available for 704 women in the HC/BPD biometry cohort from SMRU antenatal records, and a formula specific to this population was applied to estimate GA [13].

This is a retrospective analysis of clinic records. For patients who participated in trials written informed consent was obtained including consent for storage of data and samples. For the women seen at SMRU antenatal clinics, routine clinical records were anonymised and have been entered into a database since 1987. Ethical approval for audits of SMRU clinical records was given by the Oxford Tropical Research Ethics Committee (OXTREC 28–09). The original study from which the HC/BPD biometry cohort data was derived was part of the preparation and training for a fetal growth study (ClinicalTrials.gov Identifier: NCT00840502), approved by Oxford University (OxTREC (14–08)) and Mahidol University (TMEC 2008–028) Ethics Committees.

### Statistical analysis

GA estimated from Robinson and Fleming’s CRL biometry equation was used as the reference standard for GA [25,26,29]. Agreement of each method with the reference standard was determined from the mean bias and 95% limits of agreement (LOA) (calculated from the standard deviation of the mean bias), estimated using linear mixed-effects models, which are described in detail below. Birthweight-for-GA Z-scores and small for gestational age (SGA) status (Z-score <1.28 [i.e. below the 10^th^ centile]) were calculated using international centiles from the INTERGROWTH-21^st^ Project as a proxy measure of growth restriction [30]. All statistical analyses were performed in Stata Version 13 (StataCorp, College Station, Texas, US). *P*-values for all interactions were determined from likelihood ratio tests comparing models with and without interaction terms.

#### Agreement between CRL biometry and the Dubowitz method or the SFH formula

Agreement of the Dubowitz method and SFH formula was estimated using the same methods. The mean and standard deviation of the within-woman difference between methods (bias) were estimated from a linear mixed-effects model with a random-effect for the woman [31]. Interaction terms were included between method and newborn GA (centred at 39 weeks) and birthweight-for-GA Z-score to model modification of agreement, first using CRL estimates of GA and then using Dubowitz and SFH formula estimates of GA.

#### Agreement between CRL biometry and HC or BPD biometry

Agreement of HC biometry and BPD biometry measured between 16 and 40 weeks gestation were estimated using the same methods. The mean and standard deviation of the within-woman difference between methods (bias) were calculated from the estimated variance components derived from a linear mixed-effects model [31]. As HC and BPD measurements were taken twice (i.e. replicate measurements), a method by woman random effect was included and separate estimates of the residual variance were calculated for each method [31]. The resulting limits of agreement predict the accuracy of a single future HC or BPD measurement, rather than the average of two HC or BPD measurements. Interaction terms were included between method and newborn GA (centred at 39 weeks), GA at HC/BPD measurement (centred at 25 weeks), and birthweight-for-GA Z-score to model modification of agreement, first using CRL estimates of GA and then using HC and BPD estimates of GA.

#### Classifying preterm birth

To determine the accuracy of the Dubowitz method, the SFH formula, and HC or BPD biometry (measured at <25 weeks and ≥25 weeks gestation) in classifying preterm birth, % agreement, Kappa statistic, and sensitivity and specificity were calculated, using CRL biometry as the reference standard.

#### Sub-group analysis

We also determined the agreement between CRL and the Dubowitz method across newborn GA estimated from CRL biometry in pregnancies not exposed to malaria and without over-sampling of pre- and post-term newborns in concordance with the inclusion and exclusion criteria of the HC/BPD biometry cohort (N = 147).

## Results

Maternal weight in this population was relatively low, and SGA (a proxy for intrauterine growth restriction) was relatively common (Table 1). The cohorts used to determine the accuracy of the Dubowitz method and ultrasound after 14 weeks in estimating GA were different on several counts, which is unsurprising given the differences in sampling (Table 1). Importantly, the Dubowitz method cohort disproportionately selected pre- and post-term newborns, and the HC/BPD biometry cohort excluded pregnancies that were complicated by malaria (Table 1). Overlays of the distributions of newborn GA estimated from each method indicate overestimation of GA by the Dubowitz method, the SFH formula, and BPD biometry, and underestimation of GA by HC biometry in reference to CRL biometry estimates (S1 Fig).

**Table 1:** Characteristics of mother-newborn pairs in the Dubowitz method (July 2001—May 2006) and HC/BPD biometry (April 2007—October 2010) cohorts.

Variable	Dubowitz method (N = 250)	HC/BPD biometry (N = 975)
Malaria^#^	52 (21)	0 (0)
Newborn GA (CRL), weeks	38.6 {36.5–39.7}, 28.7–43.3	39.4 {38.5, 40.1}, 28.4–44.4
Very preterm (<34 weeks)	22 (9)	22 (3)
Preterm (34–36 weeks)	48 (19)	49 (5)
Term (37–41 weeks)	175 (70)	895 (92)
Post-term (≥42 weeks)	5 (2)	9 (1)
Birthweight, grams*	2722 [532], 1400–4050	3015 [420], 1210–5080
Low birthweight (<2500 grams)	88 (35)	82 (8)
Small for gestational age (<10^th^ centile)	51 (22)	175 (18)
Severe anaemia at delivery	0 (0)	0 (0)
Current smoker (yes)	74 (30)	58 (6)
Newborn’s sex (female)*	116 (47)	486 (51)
Gravidity	2 {1–4}, 1–13	2 {1–4}, 1–14
Primigravidae	71 (28)	329 (34)
Maternal age, years	25 {20–29}, 15–42	25 {21–30}, 14–47
Maternal weight at first consultation, kg	46 {43–50}, 30–68	47 {44–53}, 31–83
Population		
Refugee	236 (94)	975 (100)
Migrant	14 (6)	0 (0)

Numbers are mean [SD], range or median {inter-quartile range}, range or number (%). GA: gestational age. CRL: crown-rump length. HC: head circumference. BPD: biparietal diameter. Malaria: at least one positive smear during pregnancy and/or prior to gestational age assessment. Severe anaemia at delivery: haematocrit <20%.

^#^Malaria prior to estimation of gestational age from either the Dubowitz gestational age assessment or HC/BPD biometry measurement.

*20 missing values for birthweight and newborn sex in HC/BPD biometry cohort. 19 missing values and 25 missing values for small for gestational age in Dubowitz cohort and HC/BPD biometry cohort, respectively, due to GA limits in Z-score equations or missing birthweight.

### Agreement with CRL biometry across newborn GA and birthweight-for-GA Z-score estimated from CRL biometry

Linear mixed-effects models were fitted to determine the level of agreement between CRL biometry and the Dubowitz method, SFH formula, and HC/BPD biometry in estimating newborn GA. Where agreement was modified by one or more of newborn GA, birthweight-for-GA Z-score, or gestation time of ultrasound biometry (all calculated from CRL biometry estimates of GA), interaction parameters were included in the final models, which were centred at 39 weeks for newborn GA, 0 for birthweight-for-GA Z-score, and 25 weeks for gestation time of HC/BPD measurement.

#### The Dubowitz method

**Fig 1:**
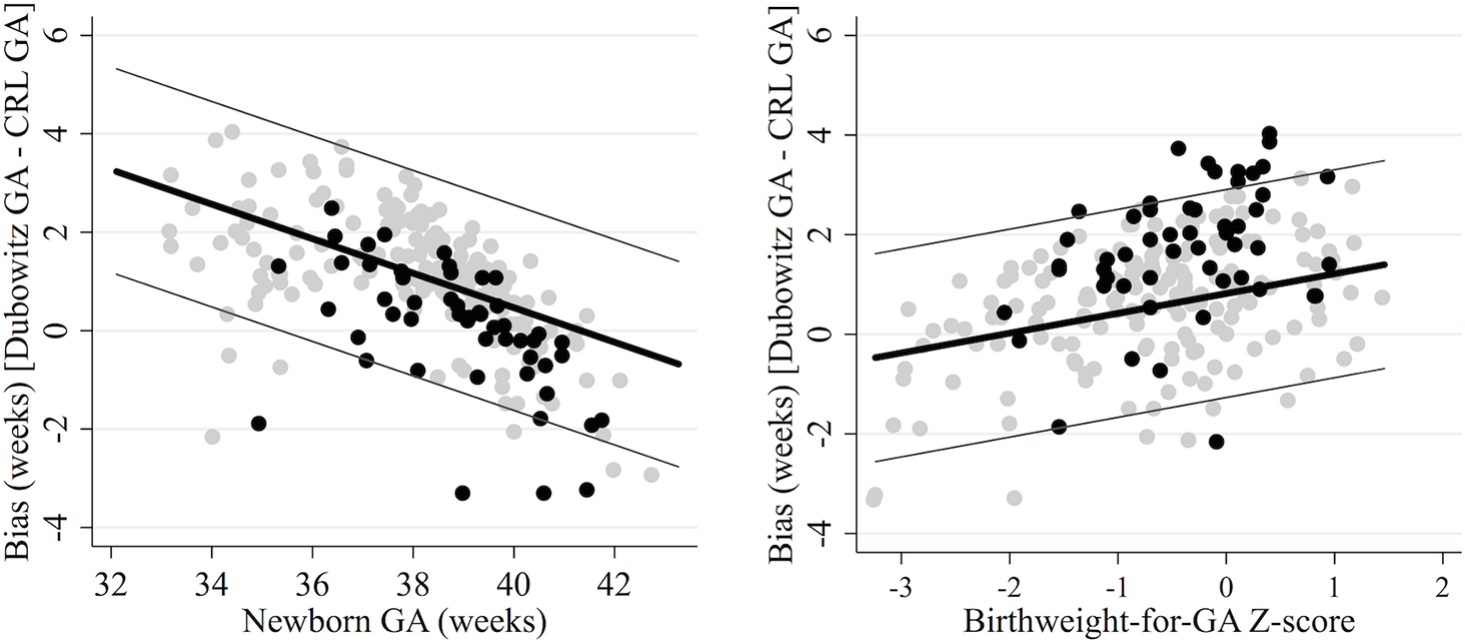
Agreement between CRL biometry and the Dubowitz method. GA: gestational age. Reference standard: crown-rump length (CRL) biometry. True gestational age determined from CRL biometry. The thick black lines represent the mean bias of the Dubowitz method in reference to CRL biometry; the thin grey lines represent the 95% limits of agreement. Grey dots are observed values for newborns with normal birthweight for GA (left) or term newborns (right); black dots are observed values for SGA newborns (left) or preterm newborns (right).

The Dubowitz method overestimated newborn GA by 2.57 weeks for a preterm newborn of 34 weeks gestation with a birthweight-for-gestational-age Z-score of 0 (95% limits of agreement (LOA): 0.49, 4.65; standard deviation (SD) = 1.04). However, mean bias decreased by 0.35 weeks per week increase in newborn GA (95% CI: -0.42, -0.28; *p* value for interaction <0.001), and increased by 0.40 weeks per unit increase in Z-score (95% CI: 0.25, 0.54; *p* value for interaction <0.001) (Fig 1). Therefore, for a newborn of 34 weeks gestation and a Z-score of -2.0 (i.e. preterm and SGA) the Dubowitz method performed slightly better, overestimating newborn GA by 1.77 weeks (95% LOA: -0.35, 3.85). For a term newborn of 40 weeks gestation the Dubowitz method performed well, even for SGA newborns, overestimating newborn GA by just 0.47 weeks if its Z-score was 0 (95% LOA: -1.62, 2.55), and underestimating by just 0.33 weeks if its Z-score was -2.0 (95% LOA: -2.41, 1.75).

#### The SFH formula

**Fig 2:**
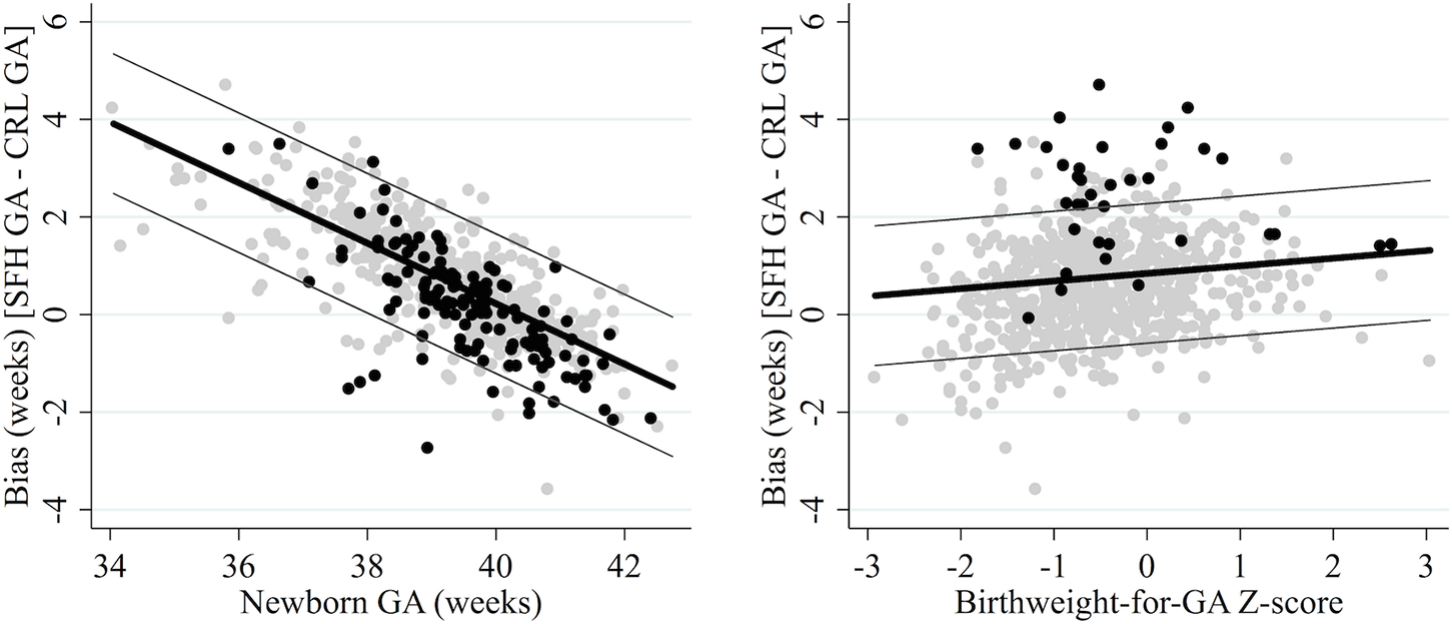
Agreement between CRL biometry and the SFH formula. Reference standard: crown-rump length (CRL) biometry. SFH: symphysis-fundal height. True gestational age determined from CRL biometry. Thick black lines represent the mean bias of the SFH formula in reference to CRL biometry; the thin grey lines represent the 95% limits of agreement. Grey dots are observed values for newborns with normal birthweight for GA (left) or term newborns (right); black dots are observed values for SGA newborns (left) or preterm newborns (right).

The SFH formula overestimated newborn GA by 3.94 weeks for a preterm newborn of 34 weeks gestation with a Z-score of 0 who had at least three SFH measurements (95% LOA: 2.50, 5.38; SD = 0.72). However, mean bias decreased by 0.62 weeks per week increase in newborn GA (95% CI: -0.66, -0.58; *p* value for interaction <0.001), and increased by 0.16 weeks per unit increase in Z-score (95% CI: 0.09, 0.22; *p* value for interaction <0.001) (Fig 2). Therefore, for a newborn of 34 weeks gestation and a Z-score of -2.0 (i.e. preterm and SGA), the SFH formula performed slightly better, overestimating newborn GA by 3.62 weeks (95% LOA: 2.18, 5.06). For a term newborn of 40 weeks gestation with a Z-score of 0 the SFH formula performed well, even for SGA newborns, overestimating newborn GA by just 0.22 weeks if its Z-score was 0 (95% LOA: -1.21, 1.65), and underestimating by just 0.10 weeks if its Z-score was -2.0 (95% LOA: -1.54, 1.34) (Fig 2).

#### HC or BPD biometry

**Fig 3:**
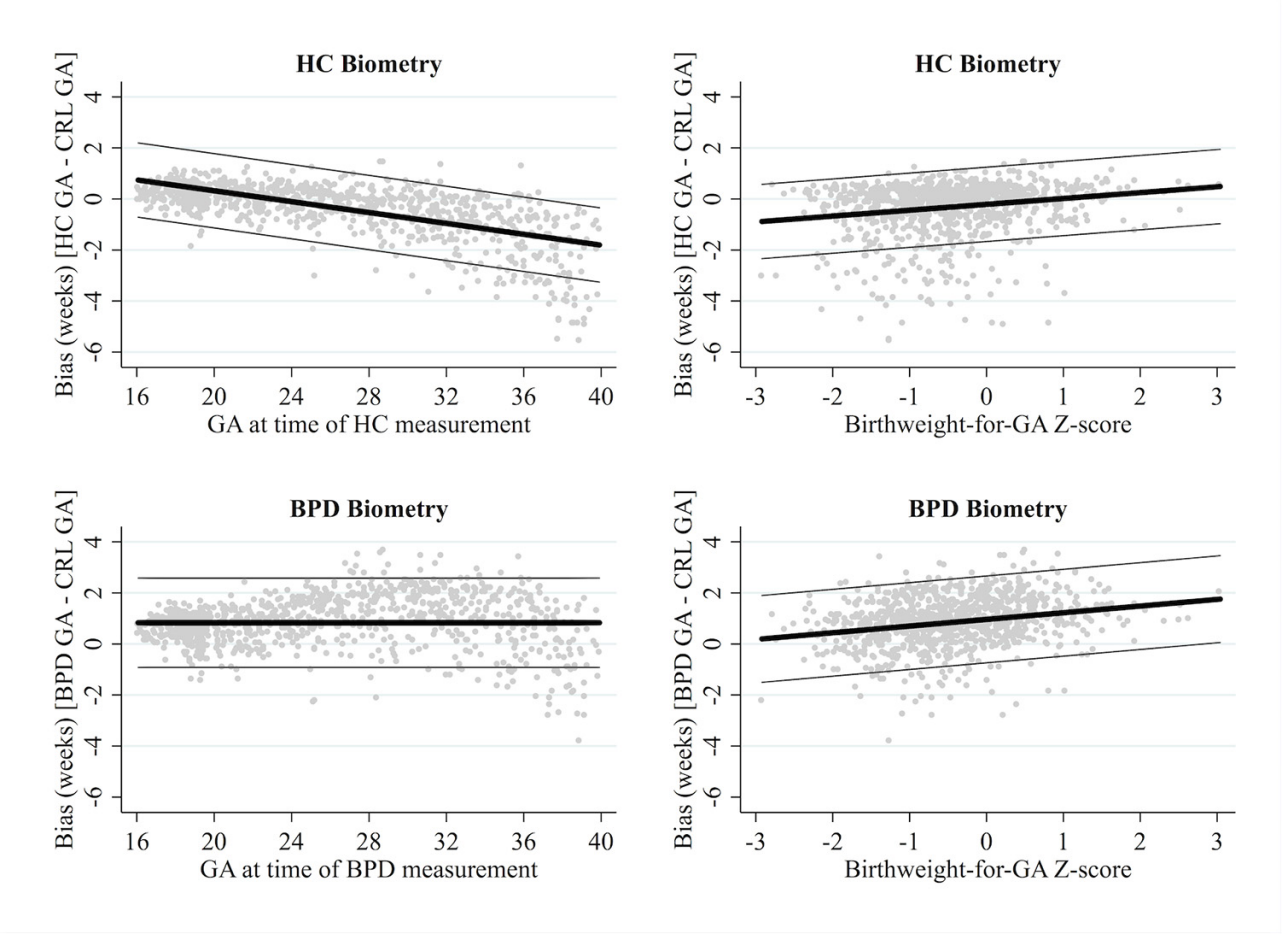
Agreement between CRL and HC or BPD biometry. Reference standard: crown-rump length (CRL) biometry. HC: head circumference. BPD: biparietal diameter. Gestation time of HC/BPD measurement determined from CRL biometry. Thick black lines represent the mean bias of HC biometry in reference to CRL biometry; the thin grey lines represent the 95% limits of agreement.

HC biometry tended to underestimate GA, especially when measured later in pregnancy, while BPD tended to overestimate GA regardless of the gestation time of measurement. On average, HC biometry underestimated GA by 0.39 weeks (95% LOA: -2.60, 1.82), however agreement was modified by gestation time of measurement and birthweight-for-GA Z-score. Mean bias decreased by 0.11 weeks per week increase in gestation time of HC measurement (95% CI: -0.11, -0.10; *p* for interaction <0.001), and increased by 0.23 weeks per unit increase in Z-score (95% CI: 0.18, 0.28; *p* for interaction <0.001) (Fig 3). When measured at 16 weeks gestation, HC biometry was more accurate in SGA newborns, slightly overestimating GA by 0.75 weeks if Z-score was 0 (95% LOA: -0.71, 2.20; SD = 0.73), but overestimating by just 0.23 weeks if Z-score was -2.0 (i.e. SGA) (95% LOA: -1.17, 1.75). However, when measured at 40 weeks gestation, HC biometry was less accurate in SGA newborns, underestimating GA by 1.81 weeks if Z-score was 0 (95% LOA: -3.27, -0.35), but by 2.27 weeks if Z-score was -2.0 (95% LOA: -3.73, -0.81).

On average, BPD biometry overestimated GA by 0.83 weeks (95% LOA: -0.93, 2.58). However, agreement was modified by birthweight-for-GA Z-score, whereby mean bias increased by 0.26 per unit increase in Z-score (95% CI: 0.21, 0.32; *p* for interaction <0.001) (Fig 3). BPD biometry was more accurate in SGA newborns, slightly overestimating GA by 0.44 weeks for a newborn with a Z-score of -2.0 (i.e. SGA) (95% LOA: -1.26, 2.14; SD = 0.85), but overestimating by 0.96 weeks for a newborn with a Z-score of 0 (i.e. not growth restricted) (95% LOA: -0.74, 2.66). For both HC biometry and BPD biometry, modification of agreement over newborn GA was not clinically significant (HC: change per week increase in newborn GA = -0.01, *p* for interaction 0.497; BPD: change per week increase in newborn GA = -0.04, *p* for interaction 0.039).

### Preterm classification

To determine the extent of misclassification that would arise due to biases associated with the Dubowitz method, the SFH formula, and HC or BPD biometry in estimating GA, we calculated agreement between methods in classifying preterm birth.

#### The Dubowitz method

The prevalence of preterm birth according to CRL biometry and the Dubowitz method was 28% (95% CI: 22, 34) and 18% (95% CI: 13, 23), respectively (Table 2). There was moderate agreement in preterm classification by the Dubowitz method in reference to CRL biometry (Kappa = 0.68) (Table 2). However, the general overestimation of GA by the Dubowitz method resulted in poor sensitivity for preterm classification (sensitivity 61%; specificity 99%) (Table 2), and misclassification of 39% (95% CI: 40, 65) of preterm newborns as term.

**Table 2:** Agreement between methods for preterm classification.

Cohort	Method	Preterm	Kappa	Sensitivity	Specificity
Dubowitz method, N = 250	CRL	70 (28)	Ref.	Ref.	Ref.
	Dubowitz	45 (18)	0.68	61 (49, 73)	99 (96, 100)
SFH formula, N = 704	CRL	42 (6)	Ref.	Ref.	Ref.
	SFH formula	13 (2)	0.31	21 (10, 37)	99 (98, 100)
HC/BPD biometry (16–24 weeks), N = 512	CRL	39 (8)	Ref.	Ref.	Ref.
	HC	35 (7)	0.80	77 (61, 89)	99 (98, 100)
	BPD	29 (6)	0.75	67 (50, 81)	99 (98, 100)
HC/BPD biometry (25–40 weeks), N = 463	CRL	32 (7)	Ref.	Ref.	Ref.
	HC	100 (22)	0.41	97 (84, 100)	84 (80, 87)
	BPD	26 (6)	0.52	50 (32, 68)	98 (96, 99)

Numbers are prevalence (%), Kappa statistic, or % sensitivity/specificity (95% Confidence Interval). Classification of preterm newborns from ultrasound HC/BPD is based on the average newborn EGA from replicate measures. Reference: preterm classification according to CRL biometry. Gestation time of HC/BPD measurement estimated from CRL biometry.

#### The SFH formula

In those with at least three SFH measurements in the HC/BPD biometry cohort, the prevalence of preterm birth according to CRL biometry and the SFH formula was 6% (95% CI: 4, 8) and 2% (95% CI: 1, 3), respectively (Table 2). There was poor agreement in preterm classification by the SFH formula in reference to CRL biometry (Kappa = 0.31) (Table 2). The general overestimation of GA by the SFH formula resulted in very poor sensitivity for preterm classification (sensitivity 21%; specificity 99%) (Table 2), and misclassification of 79% (95% CI: 63, 90) of preterm newborns as term.

#### HC or BPD biometry

The prevalence of preterm birth according to CRL biometry in the HC/BPD biometry cohort was 8% (95% CI: 6, 9). For HC and BPD biometry measured before 25 gestation weeks, preterm prevalence was 7% (95% CI: 5, 9) and 6% (95% CI: 4, 8), respectively (Table 2). Both HC and BPD measured before 25 gestation weeks achieved moderate agreement with CRL biometry (Kappa = 0.80 and 0.75 respectively), and very high specificity (99%) but average sensitivity (HC: 77%; BPD 67%) (Table 2). When measured after 25 weeks gestation, HC biometry vastly overestimated preterm prevalence (22%) and agreement for preterm classification was poor (Kappa = 0.41). BPD biometry measured after 25 weeks gestation also achieved poor agreement for preterm classification (Kappa = 0.52) (Table 2). Furthermore, BPD biometry (regardless of gestation time of measurement) and HC biometry measured after 25 weeks gestation resulted in considerable misclassification; 16% of term newborns were misclassified as preterm using HC biometry, and 40% of preterm newborns were misclassified as term using BPD biometry. However, preterm misclassification was negligible using HC biometry measured before 25 weeks gestation (1%).

### Predicting accuracy in the absence of CRL biometry

To be able to predict the accuracy of the Dubowitz method, the SFH formula, and HC or BPD biometry in practice, we also determined agreement with ultrasound CRL (reference standard) from linear mixed-effects models, with modification of agreement across variables that are known in the absence of CRL biometry.

#### The Dubowitz method

The Dubowitz method overestimated GA by 0.52 weeks for a newborn of 39 weeks gestation and a Z-score of 0 (95% LOA: -2.16, 3.30; (SD) = 1.34). Mean bias decreased by 0.29 weeks per unit increase in Z-score (calculated using Dubowitz estimates of GA) (95% CI: -0.48, -0.11; *p* for interaction = 0.002), and increased by 0.08 weeks per week increase in newborn GA (estimated using the Dubowitz method) (95% CI: -0.01, 0.18; *p* for interaction = 0.074) (Table 3). Therefore, when newborn GA was estimated at 39 weeks using the Dubowitz method, the degree of overestimation was greater for SGA newborns, overestimating GA by 1.10 weeks if Z-score was -2.0 (95% LOA: -1.58, 3.78), while agreement was similar across newborn GA. Mean bias and 95% LOAs at any Z-score and any Dubowitz estimated newborn GA (within the range of observed values in this cohort; i.e. Z-score -3.0 to 1.3 and newborn GA 32 to 42 weeks) can be calculated from these model parameters (Table 3).

**Table 3:** Parameters of linear mixed-effects models of agreement between CRL biometry and the Dubowitz method, the SFH formula, and HC or BPD biometry in estimating newborn gestational age.

	Dubowitz Model		SFH Model		HC Model		BPD Model	
Parameter	Value	SD	Value	SD	Value	SD	Value	SD
Mean bias (centred) (β_0_)	0.52 [-2.16, 3.20]	1.34	0.16 [-1.96, 2.28]	1.06	-0.37 [-1.75, 1.01]	0.69	0.39 [-1.07, 1.84]	0.73
Change in bias per unit increase in Z-score (β_1_)	-0.29 (-0.48, -0.11)	-	-0.35 (-0.44, -0.26)	-	-0.07 (-0.12, -0.03)	-	-0.11 (-0.15, -0.06)	-
Change in bias per week increase in newborn GA (β_2_)	0.08 (-0.01, 0.18)	-	0.20 (0.11, 0.29)	-	0.28 (0.25, 0.30)	-	0.30 (0.27, 0.32)	-
Change in bias per week increase in GA at ultrasound (β_3_)	-	-	-	-	-0.07 (-0.08, -0.06)	-	-0.02 (-0.02, -0.01)	-

Dubowitz and SFH models account for modification of agreement over birthweight-for-GA Z-score calculated using Dubowitz or SFH estimates of GA (mean-centred at 0) and estimated newborn GA (centred at 39 weeks). HC and BPD models account for modification of agreement over birthweight-for-GA Z-score calculated using HC/BPD estimates of GA (centred at 0), estimated newborn GA (centred at 39 weeks) and estimated gestation time of ultrasound measurement (centred at 25 weeks). Units are weeks for all values. [] – 95% limits of agreement. () – 95% confidence intervals. SD: standard deviation. These parameters can be used to calculate bias and limits of agreement in the absence of ultrasound CRL using the equations below:
$$\begin{array}{l} Bias_{Dubowitz/SFH}(95\%\;LOA)=\left[\beta_{0}+\beta_{1}\left(Zscore\right)+\beta_{2}\left(newborn\;GA-39\right)\right]\pm \left(2\times SD\right)\\ Bias_{HC/BPDbiometry}(95\%\;LOA)=\left[\beta_{0}+\beta_{1}\left(Zscore\right)+\beta_{2}\left(newborn\;GA-39\right)+\beta_{3}\left(GA\;at\;ultrasound-25\right)\right]\pm \left(2\times SD\right) \end{array}$$

#### The SFH formula

The SFH formula overestimated GA by 0.16 weeks for a newborn of 39 weeks gestation and a Z-score of 0 (95% limits of agreement (LOA): -1.96, 2.28; SD = 1.06). Mean bias decreased by 0.35 weeks per unit increase in Z-score (calculated using SFH formula estimates of GA) (95% CI: -0.44, -0.26; *p* for interaction <0.001), and increased by 0.20 per week increase in newborn GA (estimated using the SFH formula) (95% CI: 0.11, 0.29; *p* for interaction <0.001) (Table 3). Therefore, when newborn GA was estimated at 34 weeks (i.e. preterm) using the SFH formula, newborn GA was underestimated and accuracy was greater for SGA newborns: GA was underestimated by 0.14 weeks if Z-score was -2.0 (95% LOA: -2.26, 1.98), but by 0.84 weeks if Z-score was 0 (95% LOA: -2.92, 1.24). However, when newborn GA was estimated at 40 weeks (i.e. term) using the SFH formula, newborn GA was overestimated and accuracy was less for SGA newborns: GA was overestimated by 1.06 weeks if Z-score was -2.0 (95% LOA: -1.02, 3.14), but by just 0.36 weeks if Z-score was 0 (95% LOA: -1.72, 2.44). Mean bias and 95% LOAs at any Z-score and any SFH formula estimate of GA (within the range of observed values in this cohort; i.e. Z-score -3.0 to 3.2 and GA 33 to 42 weeks) can be calculated from these model parameters (Table 3).

#### HC or BPD biometry

Agreement of both HC and BPD biometry was modified by newborn GA, gestation time of measurement (estimated from HC/BPD biometry), and birthweight-for-GA Z-score (calculated from HC/BPD biometry estimates of GA) (*p* values <0.001). Therefore, our final models include interaction parameters between method and estimated newborn GA (centred at 39 weeks), estimated gestation time of measurement (centred at 25 weeks), and Z-score (centred at 0). HC biometry underestimated newborn GA by 0.37 weeks for a newborn of 39 weeks gestation with a Z-score of 0 whose HC was measured at 25 weeks gestation (95% LOA: -1.75, 1.01; SD = 0.69) (Table 3). Mean bias decreased by 0.07 weeks per one-unit increase in Z-score (95% CI: -0.12, -0.03), increased by 0.28 weeks per week increase in estimated newborn GA (95% CI: 0.25, 0.30), and decrease by 0.07 weeks per week increase in estimated gestation time of measurement (95% CI: -0.08, -0.06) (Table 3). Therefore, the degree of underestimation by HC biometry was less for a SGA newborn, underestimating by just 0.23 weeks for a newborn of 39 weeks gestation with a Z-score of -2.0 whose HC biometry was measured at 25 weeks (95% LOA: -1.61, 1.15). HC biometry measured at 16 weeks gestation for a newborn of 39 weeks and Z-score of 0 slightly overestimated GA by 0.26 (95% LOA: -1.12, 1.64) weeks, but significantly underestimated GA by -1.28 weeks if HC is measured at 38 weeks gestation (95% LOA: -2.66, 0.10). When HC biometry is measured at 25 weeks gestation and Z-score is 0, mean bias associated with HC biometry for a newborn of 34 weeks estimated from HC biometry (i.e. preterm) was -1.77 weeks (95% LOA: -3.15, 0.39), but reduces to –0.09 weeks (95% LOA: -1.47, 1.29) for a newborn of 40 weeks (i.e. term).

BPD biometry overestimated newborn GA by 0.39 weeks for a newborn of 39 weeks gestation with a Z-score of 0 whose BPD was measured at 25 weeks gestation (95% LOA: -1.07, 1.84; SD = 0.73) (Table 3). Mean bias decreased by 0.11 weeks per one-unit increase in Z-score (95% CI: -0.15, -0.06), increased by 0.22 weeks per week increase in estimated newborn GA (95% CI: 0.19, 0.24), and decreased by 0.02 weeks per week increase in estimated gestation time of measurement (95% CI: -0.02, -0.01) (Table 3). Mean bias and LOAs of ultrasound HC or BPD at any estimated newborn GA and estimated gestation time of measurement can be calculated from these model parameters, within the range of observed values (i.e. Z-score between -3.0 and +3.0 using HC biometry or -3.0 and 2.2 using BPD biomerty, estimated gestation time of ultrasound between 16 and 40 weeks, and estimated newborn GA between 28 and 42 weeks) (Table 3).

### Sub-group analysis

**Fig 4:**
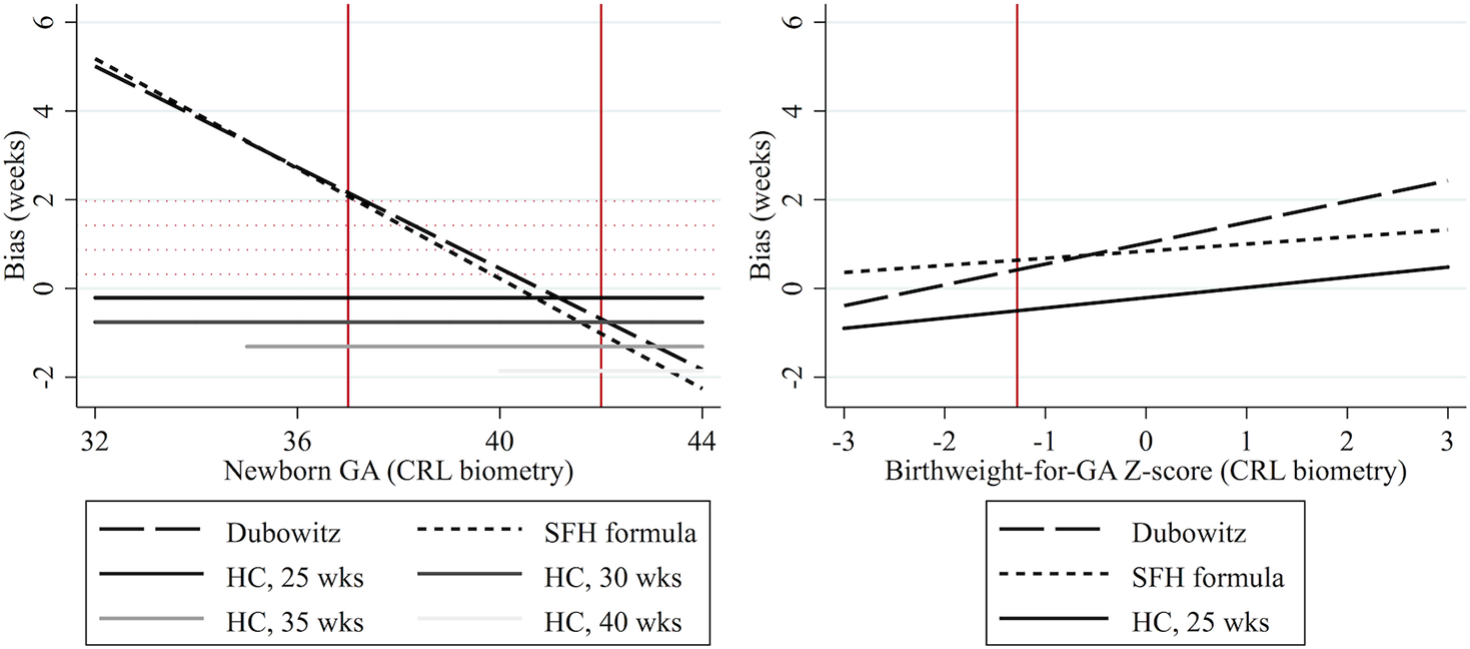
Crude comparison of biases associated with alternative methods of estimating gestational age. GA: gestational age estimated from CRL biometry. CRL: crown-rump length. SFH: symphysis fundal height. HC: head circumference, measured at 25, 30, 35 or 40 weeks gestation. Solid red vertical lines delineate cut-offs for preterm (<37 weeks) and post-term (>41 weeks) newborns, and small for gestational age (Z-score <-1.28) newborns. Dotted red horizontal lines are mirrors of HC bias to facilitate visual comparison.

In a sub-group of pregnancies not exposed to malaria and without over-sampling of pre- and post-term newborns, the Dubowitz method overestimated GA by 1.02 weeks for a newborn of 39 weeks gestation and a Z-score of 0 (95% LOA: -0.72, 2.76; SD = 0.87). Mean bias decreased by 0.52 weeks per week increase in true newborn GA (95% CI: -0.62, -0.42; *p* for interaction <0.001), and increased by 0.47 weeks per unit increase in Z-score (95% CI: 0.33, 0.62; *p* for interaction <0.001). These results can be used for a crude comparison of the relative accuracy of the Dubowitz method, SFH formula and HC biometry (Fig 4).

## Discussion

Precise estimation of GA is essential for the provision of appropriate obstetric and neonatal care, but reliance on less accurate methods for estimating GA in resource-limited settings is common. It is often forgotten that all assessments of GA are proxy markers of true GA, and all are imperfect including CRL biometry. Nevertheless, the strengths and weaknesses of each method require consideration. This study quantifies the degree of bias associated with using the Dubowitz method, the SFH formula, and HC or BPD biometry after 16 weeks gestation to estimate newborn GA with reference to CRL biometry. By modelling biases across both CRL-estimated GA and newborn parameters that are known in the absence of ultrasound CRL, our results can be used for a crude comparison of the relative accuracy of methods, and will help determine the accuracy of GA estimates in practice.

It is remarkable that the twenty-item Dubowitz GA assessment and SFH formula performed very well for term newborns, despite considerably overestimating GA of preterm newborns. These results are similar to previous studies; the Dubowitz method was reported to overestimate GA when it was first described in 1970 [15], and to a greater extent in preterm newborns [32–35], and the sensitivity of the SFH formula was shown to be poor for preterm newborns when it was first described [13]. However, we also found that bias associated with the Dubowitz method and SFH formula increased with birthweight-for-GA Z-score, which reduced the degree of overestimation in preterm SGA newborns relative to preterm newborns with normal birthweight for GA.

Second-trimester ultrasound has been shown to slightly underestimate GA depending on the biometric formula used [36–38], and the precision of GA estimates from ultrasound biometry has been shown to decrease with increasing gestation time of measurement [39,40]. However, modification of bias associated with HC or BPD biometry using the Altman & Chitty [27] and Hadlock [16] formulae, respectively, across gestation time of measurement newborn GA, and birthweight-for-GA Z-score has never been modelled. HC biometry performed well when measured at early gestations (before 25 weeks), but tended to underestimate newborn GA to a degree that increased with gestation time of measurement. BPD consistently overestimated newborn GA regardless of the gestation time of measurement. Interestingly, mean bias associated with HC and BPD biometry increased with birthweight-for-GA Z-score, which made HC biometry less accurate and BPD biometry more accurate in SGA newborns.

The tendency for HC biometry to underestimate newborn GA is unsurprising, as fetal head measurements have previously been shown to be relatively small in this population, especially later during pregnancy [1]. It was therefore unexpected that BPD biometry overestimated newborn GA, especially since BPD biometry generally has a tendency to underestimate GA for foetuses with a dolicocephalic head shape [25]. However, the accuracy of GA estimation by ultrasound biometry is highly dependent on the formula used, of which there are several [36]. BPD biometry using Hadlock’s formula has previously been shown to overestimate newborn GA in Caucasian populations, especially when measured later during pregnancy [41,42]. Our results also show that BPD biometry overestimates GA, but to a similar degree regardless of the gestation time of measurement; this may be because the accuracy of BPD biometry is also highly dependent on head shape, which varies by gestation and ethnicity [1,43].

Importantly, the ultrasound measurements used in this analysis came from a previous study on the quality of SMRU ultrasound biometry performed by locally trained health workers, and were found to be highly accurate and comparable to international standards, and SFH measurements began before 14 weeks gestation, which may limit the generalisability of our results to other resource-limited settings [1]. Additionally, maternal weight is generally low in this population, the incidence of SGA is relatively high, and there are few post-term deliveries; although we have modelled agreement over birthweight-for-GA Z-scores that were calculated using international centiles, these population characteristics may limit the generalisability of these results to populations where maternal weight is higher and SGA and preterm birth is less common. Gestational diabetes mellitus (GDM) will also influence the accuracy of gestational age estimates, however women at SMRU were not screened for GDM at this time, and a subsequent study at SMRU has shown that GDM prevalence in this population is relatively low (10%) [44].

We also modelled agreement over newborn parameters that are know in the absence of ultrasound CRL, which will help to determine the accuracy of estimates in practice. Notably, we found that bias associated with the Dubowitz method, SFH formula and HC/BPD biometry increased with birthweight-for-GA Z-score calculated using CRL biometry estimates of GA, but decreased with birthweight-for-GA Z-score calculated from Dubowitz, SFH formula, or HC/BPD biometry estimates of GA. Similarly, bias associated with the Dubowitz method and SFH formula decreased with newborn GA estimated from CRL biometry, but increased with newborn GA estimated from the Dubowitz method or SFH formula. Furthermore, the magnitude of modification of agreement across these newborn parameters differed considerably when using CRL estimates of GA compared to estimates of GA derived from alternative methods. This highlights the need to exercise caution when assessing the accuracy of GA estimates as the method used to determine GA and calculate Z-scores affects how agreement with CRL biometry is modified across these newborn parameters.

The relative accuracy of the Dubowitz method, the SFH formula, and HC biometry after 24 weeks gestation is also of clinical interest. At SMRU it is routine practice for pregnant women presenting after 24 weeks (over one third of pregnancies) to have HC biometry, SFH measurements, and a Dubowitz GA assessment available, and clinical judgement is used to determine the best estimate. Our results show that for term newborns, there is no clear difference in accuracy, except that the Dubowitz method and the SFH formula have a tendency to overestimate GA while HC biometry has a tendency to underestimate GA (Fig 4). Additionally, HC biometry allows for GA to be determined antenatally, which is important for provision of appropriate obstetric care. However, for preterm newborns, both the Dubowitz method (estimated in a sub-group analysis to account for differences between cohorts) and the SFH formula overestimate GA considerably and to a similar degree, so HC biometry should be used for the best estimate of GA in these cases, regardless of gestation time of measurement, though the degree of underestimation will be greater if growth has been restricted (Fig 4); this knowledge is of particular significance for newborns on the cusp of viability. Where ultrasound is not available, the SFH formula allows for gestation to be estimated antenatally once three SFH measurements have been recorded using an online calculator (http://www.tropmedres.ac/gestational-age), and is therefore at an advantage over the Dubowitz GA assessment despite similar agreement, especially since SFH measurements are already routinely collected in most settings. Further studies should perform ultrasound after 14 weeks, the Dubowitz Gestational Age Assessment, and SFH measurement beginning from 24 weeks gestation in the same woman for a more robust comparison of methods.

We showed that ultrasound biometry before 24 weeks gestation performs well for preterm classification. However, the Dubowitz method, the SFH formula, and to a lesser extent ultrasound biometry after 24 weeks gestation, leads to significant preterm misclassification. This misclassification is likely to be similar for other birth outcomes that are dependent on GA cut-offs, including spontaneous abortion (<28 weeks gestation in resource limited settings), stillbirth (≥28 weeks gestation), small for gestational age (<10^th^ percentile), and post-term births (>41 weeks gestation), and must be considered when estimating associations between exposures during pregnancy and adverse birth outcomes in epidemiological research [45].

Bias associated with estimating GA is critically important around the limits of viability where decisions must be made regarding the administration or withholding of intensive care and for the provision of antenatal corticosteroids during preterm labour [4,46]. By quantifying biases associated with methods used in the absence of ultrasound CRL before 14 weeks gestation, our results provide guidance regarding the level of confidence that can be conferred to GA estimates and highlight the limitations of using these methods to estimate the GA of preterm and/or growth-restricted newborns [4].

Resource-limited settings are also disproportionately affected by infections such as malaria, HIV and TB that require treatment with drugs that are either known to be contraindicated in first trimester, or have limited evidence of safety during pregnancy [2,3,22]. Understanding the effects of exposure to infection and treatment on the mother and fetus requires accurate estimation of GA to determine gestation time of exposure and to correctly classify birth outcomes. The trends in agreement are likely to be similar in other resource-limited settings, and though it is likely that the degree of agreement is likely to differ between settings, the methods used in this paper can be replicated elsewhere. Therefore, the results of this study will be informative in other populations and are relevant to hundreds of millions of pregnancies that occur in resource-limited settings each year, of which many are at risk of malaria and other serious infections [23,47]. This study quantifies the accuracy of alternative methods used for estimating GA, and will therefore help to inform appropriate obstetric and neonatal care including safe treatment of infection during pregnancy in resource-limited settings.

## Supporting Information

S1 Fig Distributions of newborn GA estimated from CRL biometry, the Dubowitz method, the SFH formula, and HC/BPD biometry. GA: gestational age. CRL: crown-rump length. SFH: symphysis-fundal height. HC: head circumference. BPD: biparietal diameter.
(TIF)
