# Supplementary figures and images for: Estimating Gestational Age in Late Presenters to Antenatal Care in a Resource-Limited Setting on the Thai-Myanmar Border
Source: PLoS One. 2015 Jun 26;10(6):e0131025. doi: 10.1371/journal.pone.0131025 (PMC4482646; doi:10.1371/journal.pone.0131025)

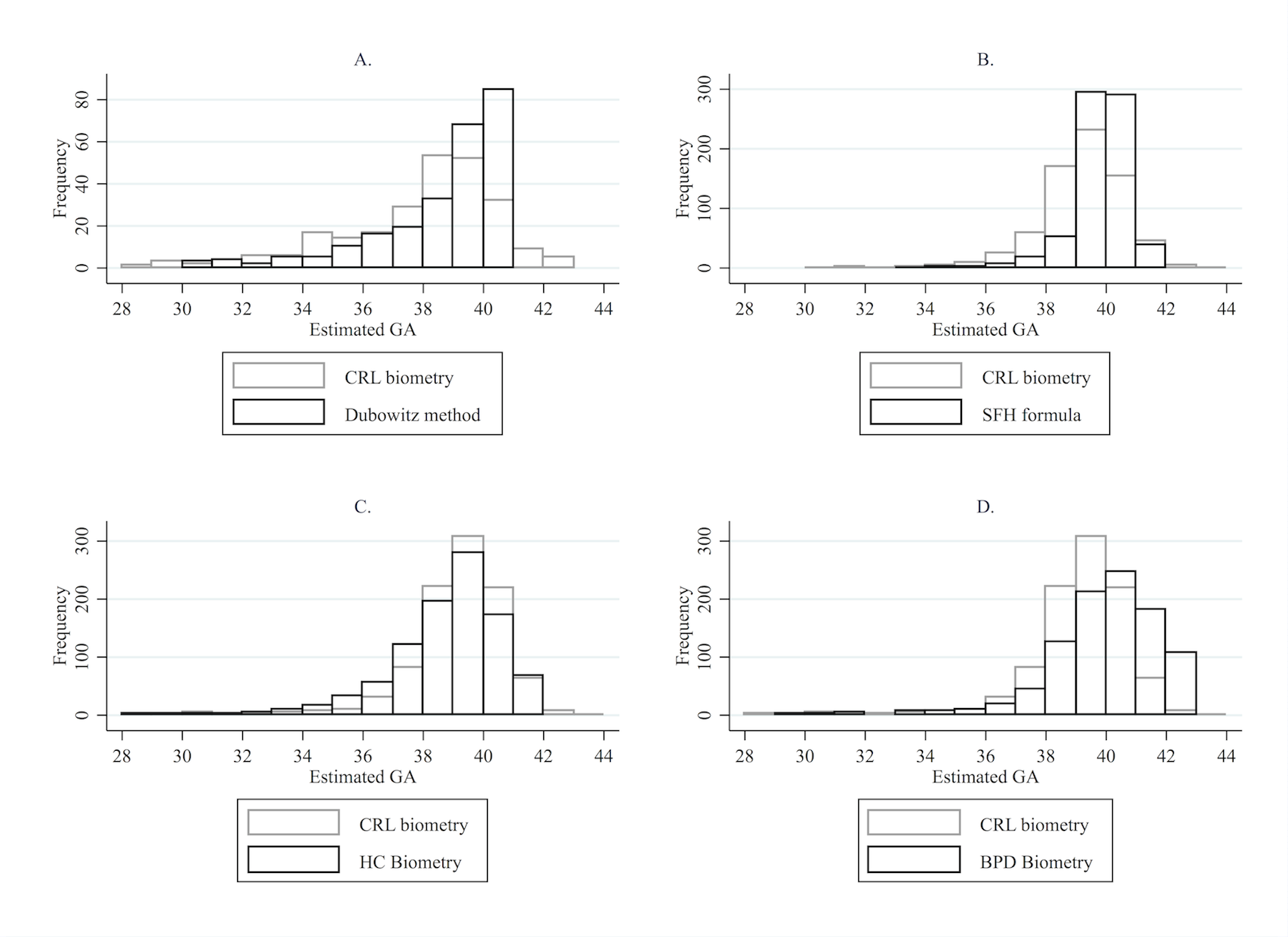

Supplement: S1 Fig — GA: gestational age. CRL: crown-rump length. SFH: symphysis-fundal height. HC: head circumference. BPD: biparietal diameter. (TIF) [file pone.0131025.s001.tif]
